# Misclassification of Hypertension Status According to Office Blood Pressure vs 24-Hour Ambulatory Blood Pressure Monitoring

**DOI:** 10.1016/j.cjco.2025.01.007

**Published:** 2025-01-11

**Authors:** Gregory L. Hundemer, Ayub Akbari, Amos Buh, Nandini Biyani, Shaafi Mahbub, Maria Salman, Pierre A. Brown, Greg A. Knoll, Manish M. Sood, Swapnil Hiremath, Marcel Ruzicka

**Affiliations:** a Division of Nephrology, Department of Medicine, University of Ottawa, Ottawa, Ontario, Canada; b Ottawa Hospital Research Institute, Ottawa, Ontario, Canada

## Abstract

**Background:**

Ambulatory blood pressure monitoring (ABPM) is the gold standard for establishing the diagnosis of hypertension yet remains underused in Canada. There remains a scarcity of Canadian data surrounding how commonly misclassification of hypertension phenotypes occurs without regular use of ABPM.

**Methods:**

This cross-sectional study included 964 consecutive adult patients referred to the Ottawa Hospital Hypertension Clinic who underwent same-day ABPM and automated office-based blood pressure measurement (AOBPM) between 2019 and 2023. The proportion of hypertension status misclassification was determined by comparing ABPM and AOBPM values. White coat hypertension (if on no antihypertensive medication) or white coat effect (if on antihypertensive medication) was defined as AOBPM ≥140/90 mm Hg but mean 24-hour ABPM <130/80 mm Hg. Masked hypertension (if on no antihypertensive medication) or masked uncontrolled hypertension (if on antihypertensive medication) was defined as AOBPM <140/90 mm Hg but mean 24-hour ABPM ≥130/80 mm Hg.

**Results:**

The mean (SD) age was 60 (16) years, and 46% of the patients were female. Among 296 patients with normotension or controlled hypertension based on ABPM, 146 (49%) met criteria for white coat hypertension (n = 21) or white coat effect (n = 125). Among 668 patients with uncontrolled hypertension based on ABPM, 364 (54%) met criteria for masked hypertension (n = 65) or masked uncontrolled hypertension (n = 299).

**Conclusions:**

The hypertension status of approximately 50% of patients was misclassified by AOBPM vs ABPM. Broader use of ABPM in Canada will improve hypertension awareness, treatment, and control rates.

## Graphical abstract

**Figure:**
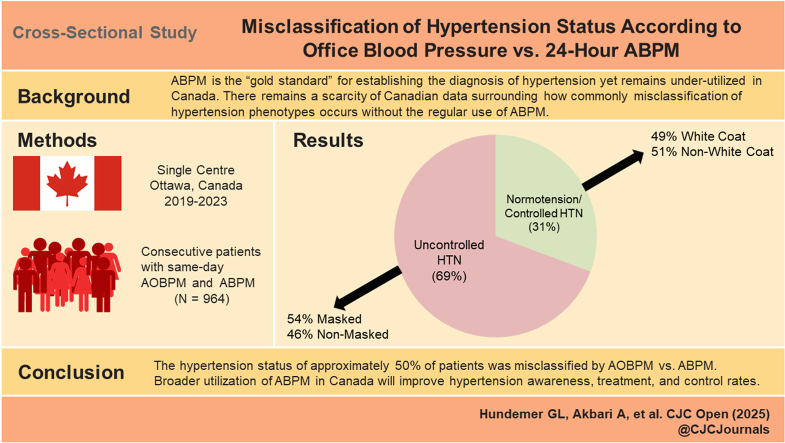

## Résumé

**Contexte:**

Le monitorage ambulatoire de la pression artérielle (MAPA) est la référence pour établir le diagnostic d’hypertension, bien qu’il demeure sous-utilisé au Canada. Les données canadiennes sont encore trop limitées pour permettre d’établir la fréquence des erreurs de classification des phénotypes de l’hypertension lorsque le MAPA n’est pas utilisé sur une base régulière.

**Méthodologie:**

Cette étude transversale comptait 964 adultes consécutifs orientés vers la clinique de l’hypertension de l’hôpital d’Ottawa qui ont été soumis, le même jour, à un MAPA et à une mesure de la pression artérielle automatisée au cabinet entre 2019 et 2023. Les erreurs sur le statut hypertensif ont été déterminées en comparant les valeurs du MAPA et celles de la mesure de la pression artérielle automatisée au cabinet. L’hypertension de consultation (en l’absence de traitement antihypertenseur) ou l’effet de la blouse blanche (en présence d’un traitement antihypertenseur) étaient définis comme une mesure de la pression artérielle automatisée au cabinet ≥ 140/90 mmHg, mais avec une valeur de MAPA moyenne sur 24 heures < 130/80 mmHg. L’hypertension masquée (en l’absence de traitement antihypertenseur) ou l’hypertension non maîtrisée masquée (en présence de traitement antihypertenseur) étaient définies comme une mesure de la pression artérielle automatisée au cabinet < 140/90 mmHg, mais avec une valeur de MAPA moyenne sur 24 heures ≥ 130/80 mmHg.

**Résultats:**

L’âge moyen (écart-type) était de 60 (16) ans; 46 % des patients étaient des femmes. Sur les 296 patients affichant une pression normale ou une hypertension maîtrisée selon le MAPA, 146 (49 %) répondaient aux critères de l’hypertension de consultation (n = 221) ou de l’effet de la blouse blanche (n = 125). Sur les 668 patients présentant une hypertension non maîtrisée selon le MAPA, 364 (54 %) répondaient aux critères d’hypertension masquée (n = 65) ou d’hypertension non maîtrisée masquée (n = 299).

**Conclusions:**

Par rapport au MAPA, la mesure de la pression artérielle automatisée au cabinet a faussé l’interprétation du statut hypertensif chez environ 50 % des patients. Une utilisation élargie du MAPA au Canada permettra de mieux connaître l’hypertension, son traitement et son taux de maîtrise.

Hypertension is the single most common and modifiable risk factor for cardiovascular disease and mortality.^1^ The prevalence of hypertension remains high, being present in approximately 1 in 4 Canadian adults. Whereas Canada has historically had high hypertension treatment and control rates relative to other nations, these rates have declined over recent years, particularly among women, of whom 35% remain untreated and 51% are uncontrolled.^2^ This has developed in spite of an ever-increasing armamentarium of blood pressure (BP)-lowering treatments being developed for clinicians to improve hypertension control.^3^

A key to improving hypertension control rates is accurate diagnosis. Historically, automated office-based BP measurement (AOBPM) has been the most used approach to make the diagnosis of hypertension. However, AOBPM may not always provide an accurate reflection of a patient’s BP. For instance, AOBPM may misclassify patients as being normotensive (ie, masked hypertension) or hypertensive (ie, white coat hypertension) when compared to home BP measurements (HBPM). Appropriately making these diagnoses is essential as they carry important long-term implications on cardiovascular disease risk.^4^, ^5^, ^6^ Moreover, the change in BP from daytime to nighttime provides prognostic information, with the average person experiencing a 10% to 20% BP decrease (ie, “dipping”) from day to night.^7^ Independent of the degree of hypertension, a <10% decrease in BP from day to night (ie, “non-dipping”) is associated with an increased risk for cardiovascular complications.^8^, ^9^, ^10^, ^11^ Extreme dipping (>20% decrease in BP from day to night) may also be detrimental although this is more controversial because of conflicting literature.^12^, ^13^, ^14^ The nocturnal BP measurements required to identify these BP trends are not captured by AOBPM or conventional HBPM. Because of these limitations with traditional BP measurement approaches, 24-hour ambulatory blood pressure monitoring (ABPM) has become widely accepted as the gold standard in establishing the diagnosis of hypertension.^15^

Nevertheless, ABPM remains heavily underused in Canada. For instance, in a survey study of Canadian family physicians, a mere 14% of respondents reported using ABPM for the diagnosis of hypertension.^16^ This low use of ABPM occurs in spite of evidence of it being cost effective within Canada.^17^ However, there is a scarcity of existing real-world data on the impact of low ABPM use on hypertension diagnoses. Herein, we present a large cross-sectional study of consecutive adult patients who underwent same-day AOBPM and ABPM at the Ottawa Hospital Hypertension Clinic to determine the proportion of hypertension misclassification and abnormal nocturnal BP patterns detected via ABPM.

## Material and Methods

### Study design

This was a cross-sectional study of consecutive adult patients who underwent ABPM at the Ottawa Hospital Hypertension Clinic (Ottawa, Ontario, Canada) between 2019 and 2023. The research reported in the article adhered to the **ST**rengthening the **R**eporting of **OB**servational studies in **E**pidemiology (STROBE) guidelines for cross-sectional studies.^18^ This study was approved by the Ottawa Health Science Network Research Ethics Board (protocol ID 20230405-01H). This is a retrospective study using deidentified data; therefore, the research ethics board did not require consent from the patients.

### Setting

This study was conducted at the Ottawa Hospital Hypertension Clinic. The Ottawa Hospital is a tertiary academic centre with a catchment area of approximately 1.3 million people. The Ottawa Hospital Hypertension Clinic is the largest hypertension specialty clinic in Canada and is the first in Canada to be designated as a “Comprehensive Hypertension Center” by the American Society of Hypertension. Patients can be referred to the clinic by health care providers from any discipline to be seen by a hypertension specialist or for ABPM alone. Therefore, ABPM requests come from an array of health care providers ranging from hypertension specialists to primary care providers.

### Study cohort

The study cohort was derived from a database of all adult (≥18 years of age) patients referred to the Ottawa Hospital Hypertension Clinic who underwent ABPM from January 1, 2019, to March 31, 2023. The database undergoes random data audits every 6 months where the data are compared with the electronic medical record to ensure accuracy. For patients who underwent multiple ABPMs, only the first report was considered for this study. Additional exclusion criteria included inadequate numbers of ABPM readings (defined as <20 daytime readings or <7 nighttime readings as per Hypertension Canada guidelines)^19^ or missing AOBPM (captured at the time of ABPM placement as per clinic protocol). AOBPM is performed using the Welch Allyn Connex Spot Monitor (Welch Allyn, Inc; Skaneateles Falls, NY). Following a 5-minute rest period, unattended BP is measured 3 times at 1-minute intervals, and the mean is reported. ABPM is performed using the Spacelabs 90227 device (Spacelabs Healthcare; Snoqualmie, WA).

### Statistical analysis

For baseline data, continuous variables were expressed as mean (standard deviation [SD]) if normally distributed and as median (25th to 75th percentile interquartile range [IQR]) if non-normally distributed, whereas categorical variables were expressed as number (percentage). Sleep quality during ABPM was assessed based on a qualitative scale that patients were asked to complete at the end of the ABPM testing with the following possible options: very good, good, average, poor, and very poor. The prevalence of hypertension phenotypes (isolated daytime hypertension, isolated nighttime hypertension, sustained hypertension, and no hypertension) was then determined. Daytime hypertension was defined as mean daytime systolic BP ≥135 mm Hg and/or mean daytime diastolic BP ≥85 mm Hg.^19^ Nighttime hypertension was defined as mean nocturnal systolic BP ≥120 mm Hg and/or mean nocturnal diastolic BP ≥70 mm Hg.^19^ Based on the ABPM results, patients were categorized as having normotension or controlled hypertension if the mean 24-hour BP according to ABPM was <130/80 mm Hg vs uncontrolled hypertension if the mean 24-hour BP according to ABPM was ≥130/80 mm Hg. Further, patients with normotension or controlled hypertension according to 24-hour ABPM were categorized based on whether they had white coat hypertension (if not prescribed antihypertensive medication) or white coat effect (if prescribed antihypertensive medication), defined by a same-day AOBPM ≥140/90 mm Hg. Patients with uncontrolled hypertension according to 24-hour ABPM were categorized based on whether they had masked hypertension (if not prescribed antihypertensive medication) or masked uncontrolled hypertension (if prescribed antihypertensive medication), defined by a same-day AOBPM <140/90 mm Hg. A secondary analysis was conducted using the same methodology but redefining the reference standard for uncontrolled hypertension as a mean daytime ABPM of ≥135/85 mm Hg. The prevalence of nocturnal BP dipping patterns was categorized as follows: normal dipping (systolic BP decrease of 10%-20%), extreme dipping (systolic BP decrease of >20%), non-dipping (systolic BP decrease of <10%), and reverse dipping (systolic BP increase).^20^

## Results

### Baseline characteristics

**Figure 1:**
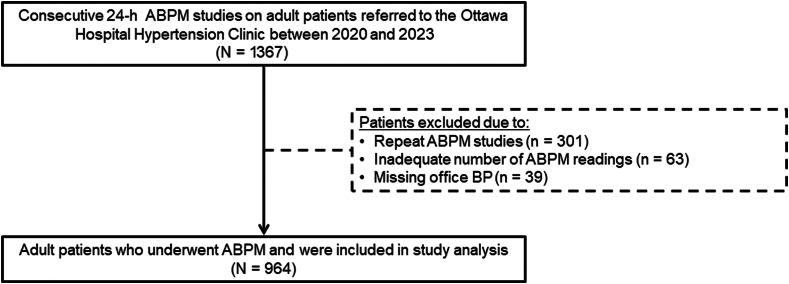
Flow diagram for study cohort. ABPM, ambulatory blood pressure monitoring; BP, blood pressure.

A total of 1367 ABPM studies were performed at the Ottawa Hospital Hypertension Clinic from January 1, 2019, to March 31, 2023 (Fig. 1). The final study cohort included 964 patients after excluding those with repeat ABPM studies (n = 301), inadequate number of ABPM readings (n = 63), and missing same-day AOBPM (n = 39). The baseline characteristics of the study cohort are displayed in Table 1. The mean (SD) age was 60 (16) years, and 46% of patients were female. The mean body mass index was 29.8 (7.7). The mean AOBPM was 139 (17)/78 (12) mm Hg. The majority of patients (83%) were prescribed antihypertensive medications with a median (IQR) of 2 (1-3) medications per patient. Approximately half of the patients reported poor (42%) or very poor (4%) sleep quality during the ABPM study.

**Table 1:** Baseline characteristics of the study cohort (N = 964)

Characteristic	Value
Age, y, mean (SD)	60 (16)
Sex, n (%)	
Female	442 (46)
Male	522 (54)
Body mass index, mean (SD)	29.8 (7.7)
Office blood pressure, mm Hg, mean (SD)	
Systolic	139 (17)
Diastolic	78 (12)
ABPM blood pressure, mm Hg, mean (SD)	
24-h systolic	130 (12)
24-h diastolic	74 (10)
Daytime systolic	134 (13)
Daytime diastolic	78 (11)
Nighttime systolic	120 (15)
Nighttime diastolic	66 (10)
Any antihypertensive medication prescribed, n (%)	797 (83)
Number of antihypertensive medications prescribed, median (IQR)	2 (1-3)
Antihypertensive medication categories, n (%)	
α-Blocker	36 (4)
ACE inhibitor	325 (34)
Angiotensin II receptor blocker	285 (30)
β-Blocker	288 (30)
Calcium channel blocker	526 (55)
Central agonists	21 (2)
ENaC inhibitor	28 (3)
Direct vasodilators	10 (1)
Loop diuretic	33 (3)
Mineralocorticoid receptor antagonist	126 (13)
Thiazide-type diuretic	328 (34)
Sleep quality, n (%)∗	
Very good	30 (4)
Good	267 (32)
Average	146 (18)
Poor	347 (42)
Very poor	34 (4)

ABPM, ambulatory blood pressure monitoring; ACE, angiotensin-converting enzyme; ENaC, epithelial sodium channel; IQR, 25th-75th percentile interquartile range; SD, standard deviation.

∗ Sleep quality data missing in 140 patients (15% of study cohort).

### ABPM hypertension phenotypes

Table 2 displays the prevalence of ABPM hypertension phenotypes along with mean daytime and nighttime BP measurements among the study cohort. The most common phenotype was sustained hypertension (43%), followed by normotension/controlled hypertension (31%), isolated nighttime hypertension (14%), and isolated daytime hypertension (12%).

**Table 2:** ABPM hypertension phenotypes among study cohort (N = 964)

	Isolated daytime hypertension (n = 120; 12%)	Isolated nighttime hypertension (n = 131; 14%)	Sustained hypertension (n = 417; 43%)	Normotension/controlled hypertension (n = 296; 31%)
Daytime systolic BP, mm Hg, mean (SD)	139 (7)	128 (6)	144 (9)	123 (9)
Daytime diastolic BP, mm Hg, mean (SD)	81 (8)	73 (9)	82 (11)	73 (8)
Nighttime systolic BP, mm Hg, mean (SD)	112 (5)	123 (9)	131 (13)	107 (8)
Nighttime diastolic BP, mm Hg, mean (SD)	62 (6)	68 (8)	71 (10)	60 (6)

ABPM, ambulatory blood pressure monitoring; BP, blood pressure; SD, standard deviation.

### White coat and masked hypertension phenotypes

**Figure 2:**
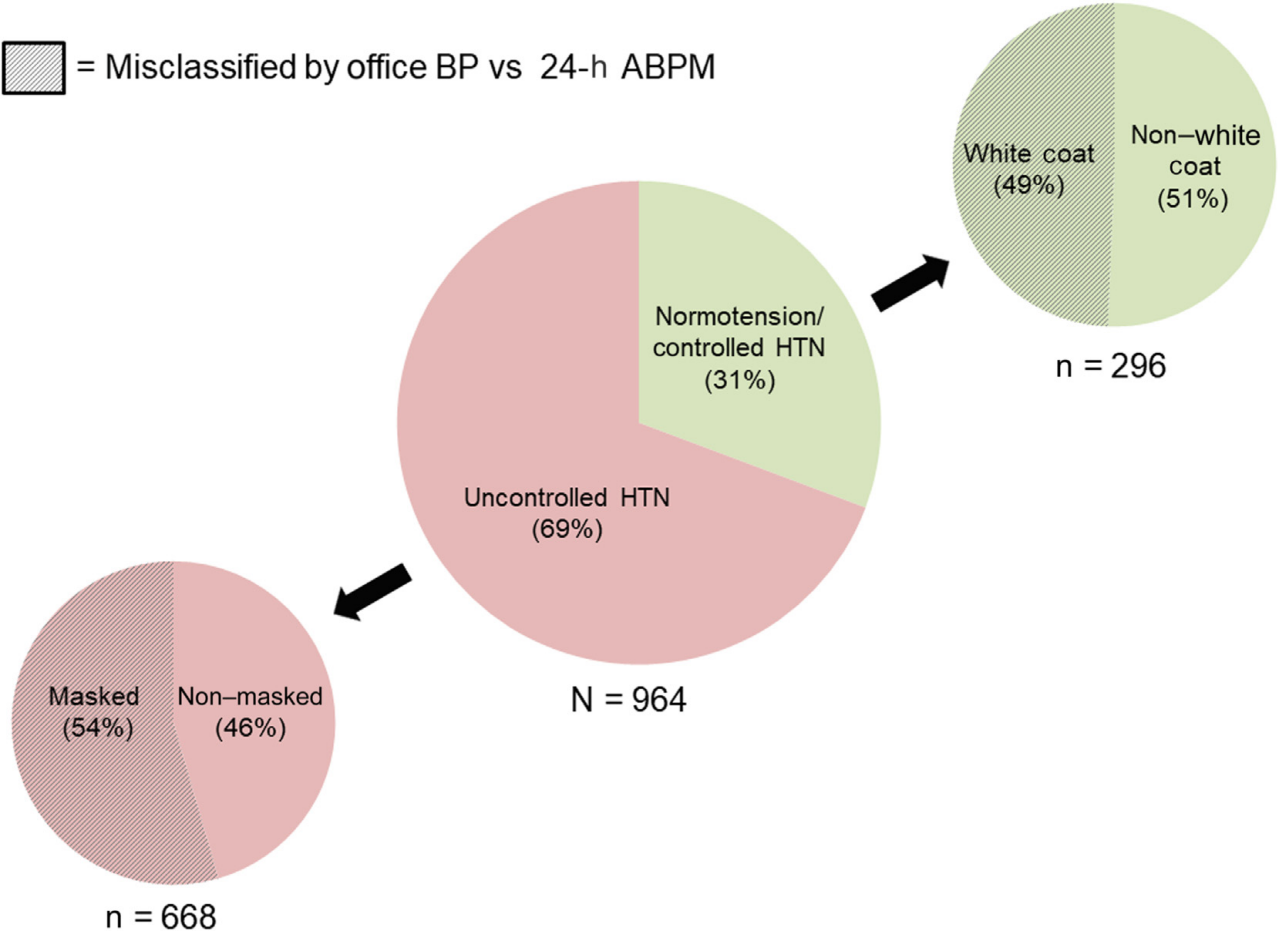
Misclassification of hypertension status with AOBPM relative to ABPM among the study cohort. Central pie chart represents the proportion of uncontrolled hypertension and normotension/controlled hypertension based on mean 24-hour ABPM ≥130/80 mm Hg or <130/80 mm Hg, respectively. White coat hypertension (if on no antihypertensive medication) or white coat effect (if on antihypertensive medication) was defined as AOBPM ≥140/90 mm Hg but mean 24-hour ABPM <130/80 mm Hg. Masked hypertension (if on no antihypertensive medication) or masked uncontrolled hypertension (if on antihypertensive medication) was defined as AOBPM <140/90 mm Hg but mean 24-hour ABPM ≥130/80 mm Hg. ABPM, ambulatory blood pressure monitoring; AOBPM, automated office-based blood pressure measurement; BP, blood pressure; HTN, hypertension.

Figure 2 displays the prevalence of white coat and masked hypertension phenotypes when comparing ABPM to AOBPM with mean 24-hour ABPM as the reference standard. Among 296 patients with normotension or controlled hypertension according to 24-hour ABPM, 146 (49%) had AOBPM ≥140/90 mm Hg and thus met criteria for white coat hypertension (21 of 167 patients prescribed no antihypertensive medication [13%]) or white coat effect (125 of 797 patients prescribed antihypertensive medication [16%]). Therefore, these patients would have been misclassified as having uncontrolled hypertension by AOBPM alone. Among 668 patients with uncontrolled hypertension according to 24-hour ABPM, 364 (54%) had AOBPM <140/90 mm Hg and thus met criteria for masked hypertension (65 of 167 patients prescribed no antihypertensive medication [39%]) or masked uncontrolled hypertension (299 of 797 patients prescribed antihypertensive medication [38%]). Therefore, these patients would have been misclassified as having normotension or controlled hypertension by AOBPM alone.

Supplemental Figure S1 displays the results from the secondary analysis where the reference standard was redefined based on mean daytime ABPM. Among 427 patients with normotension or controlled hypertension according to daytime ABPM, 146 (34%) met criteria for white coat hypertension or white coat effect. Among 537 patients with uncontrolled hypertension according to daytime ABPM, 173 (32%) met criteria for masked hypertension or masked uncontrolled hypertension.

### Nocturnal dipping patterns

**Figure 3:**
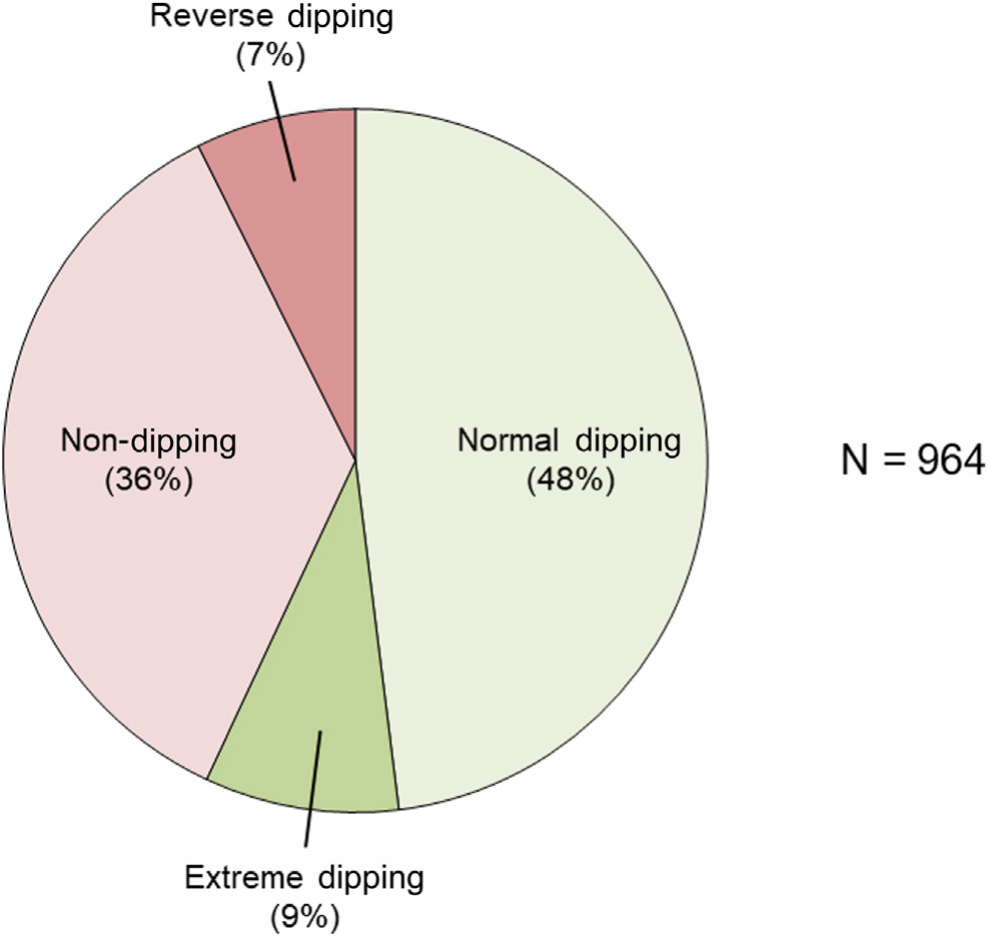
Prevalence of nocturnal BP dipping patterns among the study cohort. Nocturnal dipping patterns were categorized as follows: normal dipping (systolic BP decrease of 10%-20%), extreme dipping (systolic BP decrease of >20%), non-dipping (systolic BP decrease of <10%), and reverse dipping (systolic BP increase). BP, blood pressure.

Figure 3 displays the prevalence of nocturnal BP dipping phenotypes among the study cohort. Normal dipping was the most common phenotype present in roughly half of the cohort (48%) followed by non-dipping (36%), extreme dipping (9%), and reverse dipping (7%).

## Discussion

In this cross-sectional study of 964 consecutive adult patients who underwent ABPM at the Ottawa Hospital Hypertension Clinic, we found that the hypertension status of approximately half of all patients were misclassified based on same-day AOBPM relative to ABPM. This included 49% of patients with normotension or controlled hypertension on ABPM yet AOBPM of ≥140/90 mm Hg (ie, white coat hypertension or white coat effect). Moreover, 54% of patients with uncontrolled hypertension on ABPM had same-day AOBPM of <140/90 mm Hg (ie, masked hypertension or masked uncontrolled hypertension). Given the well-established long-term cardiovascular risk implications inherent to white coat and masked hypertension phenotypes,^4^^,^^5^ these findings highlight that an alarmingly high proportion of the Canadian population may be misclassified in regard to hypertension status given the low nationwide use of ABPM.

Abnormal nocturnal BP dipping patterns were also exceedingly common, being present in approximately half of the study cohort. Overall, 36% of patients were non-dippers (nocturnal systolic BP decrease of <10%), 7% were reverse dippers (nocturnal systolic BP increase), and 9% were extreme dippers (nocturnal systolic BP decrease of >20%). Identification of these abnormal nocturnal BP patterns is important as they are highly prognostic of increased cardiovascular risk, and their identification may provide opportunities to optimize cardiovascular risk mitigation strategies.^8^, ^9^, ^10^, ^11^ Beyond its prognostic implications, it is important to note that there is no good evidence that reversal of non-dipping is either possible or beneficial. However, the presence of non-dipping may trigger an evaluation into underlying causes such as sleep apnea or autonomic dysfunction.^21^^,^^22^ Without ABPM, these abnormal nocturnal BP patterns go completely unrecognized in clinical practice, thereby further emphasizing the negative implications of low ABPM use in Canada.

Although Canada has gained a reputation for being among the world’s leading nations in hypertension control rates,^23^ recent years have witnessed a step back in these statistics. This includes a decline in hypertension awareness, treatment, and control rates, particularly among females.^2^^,^^24^ Accurate diagnosis of hypertension is a critical first step in order to improve hypertension control rates and identify patients who are most likely to benefit from the ever-improving armamentarium of antihypertensive therapies. ABPM provides the modern-day gold standard for hypertension diagnosis by accurately identifying patients misclassified as normotensive by AOBPM (ie, masked hypertension), patients misclassified as hypertensive by AOBPM (ie, white coat hypertension), and patients with abnormal nocturnal BP patterns who are at increased cardiovascular risk independent of daytime BP.^25^^,^^26^ As evidenced by the results from the present study, these phenotypes of masked hypertension, white coat hypertension, and abnormal nocturnal dipping should not be viewed as niche diagnoses. Rather, they are common, as consistent with prevalence data from other geographic locations around the world.^27^, ^28^, ^29^, ^30^, ^31^, ^32^

These findings on hypertension misclassification based on AOBPM vs ABPM, which has not previously been well studied in Canada, confirms a highly relevant clinical gap in hypertension management at centres that do not incorporate ABPM as part of standard practice. Recommendations surrounding increased use of ABPM as the gold standard by which to assess for hypertension now exist in many major jurisdictions around the world. For instance, the United Kingdom declared ABPM as the preferred method by which to make the diagnosis of hypertension.^33^ The United States Preventive Services Task Force issued a grade A recommendation for ABPM to confirm the diagnosis of hypertension outside of the clinic setting.^34^ The recently released European Society of Cardiology and European Society of Hypertension clinical practice guidelines also recommend ABPM for confirmation of hypertension diagnosis.^35^^,^^36^ However, these recommendations have not necessarily translated into substantial increases in ABPM uptake in many settings.

The reasons behind why high rates of hypertension misclassification occur with AOBPM relative to ABPM are multifactorial. For instance, BP is inherently a labile physiologic measurement. By averaging a large number of BP measurements over a full 24-hour cycle, ABPM smooths out many of the peaks and valleys in BP lability which make AOBPM prone to error.^37^ Moreover, the doctor’s office causes stress for many patients, which can influence AOBPM even if measured in ideal settings (eg, having a series of unattended measures following a rest period averaged). ABPM better captures BP trends in a patient’s normal day-to-day environment. Timing of AOBPM relative to when antihypertensive medications are taken may further predispose to inaccuracies. Although most commonly used antihypertensive medications are long-acting, their peak effect is generally within 2-3 hours after being taken. Therefore, AOBPM values may be biased based on when a patient takes their medication while ABPM overcomes this by generating a 24-hour average. Notably, these factors (among others) may serve to explain why prior meta-analysis data show that ABPM reproducibility is excellent at the population level but limited at the intraindividual level.^38^

Several explanations for the low rates of ABPM use in Canada may be postulated. For most Canadian family care practices where the bulk of hypertension cases are diagnosed and managed, ABPM requires referral to a local hospital or noninvasive vascular testing laboratory with only approximately 20% of family care practices having ABPM available on site.^16^ This infrastructure introduces an extra layer of complexity to obtaining ABPM as it requires referral to another centre, which comes with its own inherent challenges for patients, particularly for those individuals who reside in more rural locations. Indeed, increased ABPM accessibility has been proven to increase its use.^39^ Moreover, reimbursement remains a major barrier to increase ABPM use in Canada with variable coverage across provinces.^17^ This limited reimbursement persists despite evidence of the cost effectiveness of ABPM. For example, a 2012 health economics study from Health Quality Ontario found ABPM to be cost effective with an incremental cost effectiveness ratio (ICER) of CAD$30 per quality-adjusted life year (QALY), which would translate into $19 million in cost savings to the Canadian health care system over 5 years.^17^ These health savings stem mostly from improved and faster diagnosis, thus decreasing physician visits along with reduced medication use for the diagnosis of white coat hypertension and/or white coat effect. Finally, high up-front device costs and the need to develop expertise in both performing and interpreting ABPM provide additional barriers that further limit uptake.^40^ Ultimately, a multipronged approach including (but not limited to) enhanced knowledge dissemination on the benefits of ABPM, increased accessibility in the primary care setting, improved reimbursement, subsidized device costs, and more streamlined training may be required to substantially increase ABPM use at the national level.

Arguably, HBPM is an alternative to ABPM that has become more widespread in recent years. HBPM also helps empower patients in managing their own BP, and HBPM use has been reported to be associated with improved rates of BP control.^41^ There is, indeed, high but not complete agreement between HBPM and ABPM for the diagnoses of white coat and masked hypertension.^42^ However, HBPM does not allow for assessment of BP variability and, most importantly, nocturnal dipping patterns.^43^ Lastly, HBPM devices are not always accurate, and a recent Canadian survey reported a large proportion of devices being sold to be not validated.^44^, ^45^, ^46^

We acknowledge several limitations within our study. First, this was a single-centre study, which may limit the generalizability of our findings. Our hypertension specialty clinic is unique in being one of the largest and most active ABPM centres in Canada; however, the results may not generalize to the primary care population or to other geographic locations. Second, the cohort was made up of patients referred to the Ottawa Hospital Hypertension Clinic, which may have introduced an element of selection bias. Specifically, there may have been an underlying concern for white coat or masked hypertension phenotypes in these cases as the reason for ABPM testing. For instance, among patients on no antihypertensive medications, the prevalence of white coat hypertension in our study (13%) was similar to prior studies suggesting a prevalence of 10% to 20%.^27^, ^28^, ^29^ However, the prevalence of masked hypertension in our study (39%) was higher than that of most prior studies, which have suggested a prevalence of 10% to 30%.^30^, ^31^, ^32^ This may reflect that many prior studies were based out of community-based practices as compared to our referral-based centre where there may have been a higher initial suspicion for masked hypertension, which led to the ABPM being performed. Third, the study was cross-sectional and identified only prevalent hypertension phenotypes. Therefore, we were unable to capture longitudinal outcomes in this cohort. Finally, although ABPM provides more accurate BP assessment and better correlation with future all-cause mortality and cardiovascular events,^6^^,^^47^ AOBPM is the more traditional approach used in both clinical practice and clinical trials given its sheer convenience. Whether greater use of ABPM in routine practice will lead to improved long-term health outcomes still remains an uncertain area in need of future research.

## Conclusions

The hypertension status of approximately 50% of patients who underwent ABPM at the Ottawa Hospital Hypertension Clinic was misclassified based on same-day AOBPM relative to ABPM. This included a high prevalence of masked and white coat hypertension phenotypes that were revealed via ABPM. Moreover, approximately 50% of patients also had abnormal nocturnal BP dipping patterns, which are associated with high cardiovascular risk, independent of daytime BP. These findings, along with low ABPM use rates across Canada, showcase a major missed opportunity to improve hypertension management at the population level. We strongly advocate for the more widespread use of ABPM in routine clinical practice to improve hypertension awareness, treatment, and control rates and thereby optimize long-term patient health outcomes.
