# Supplementary material for: Misclassification of Hypertension Status According to Office Blood Pressure vs 24-Hour Ambulatory Blood Pressure Monitoring
Source: CJC Open. 2025 Jan 11;7(4):508–15. doi: 10.1016/j.cjco.2025.01.007 (PMC12105752; doi:10.1016/j.cjco.2025.01.007)
Supplement: Supplemental Figure [file mmc1.pdf]

SUPPLEMENTARY MATERIAL

Misclassification of Hypertension Status According to Office Blood Pressure versus 24-Hour Ambulatory Blood Pressure Monitoring

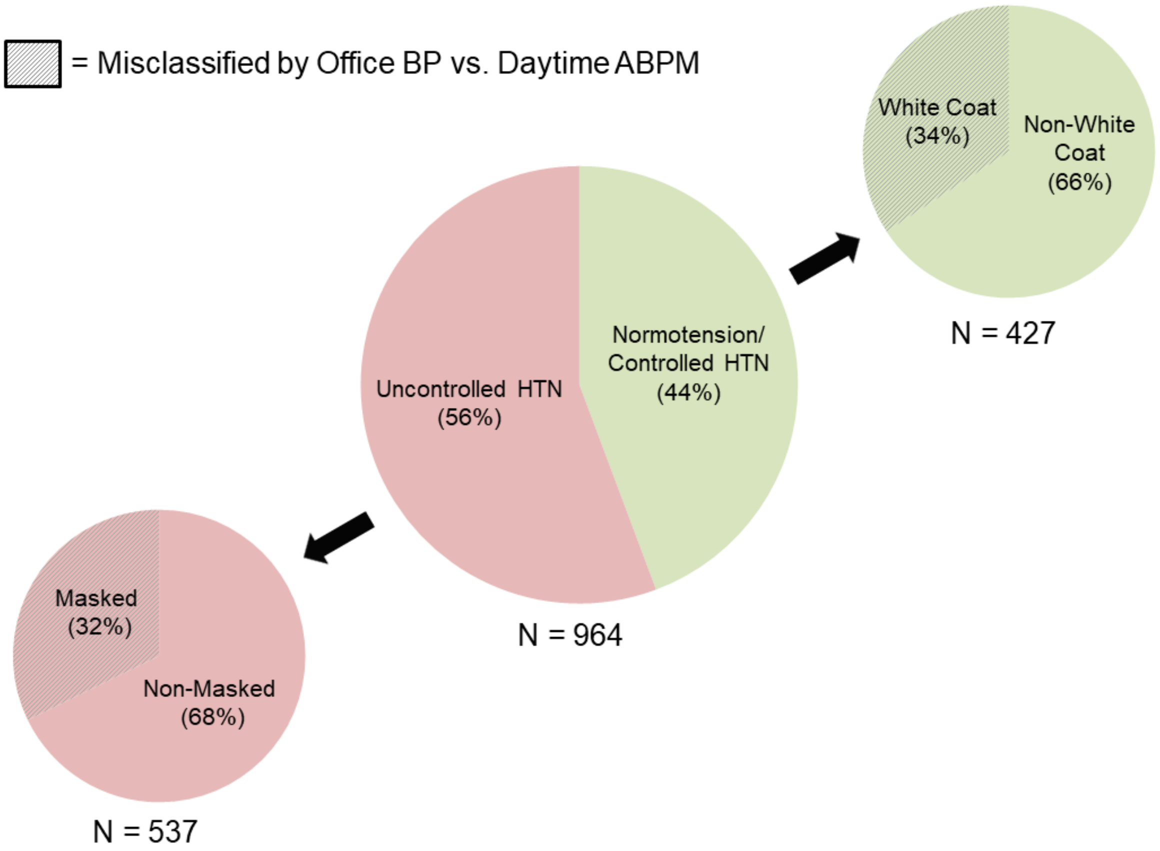

Supplemental Figure S1. Misclassification of hypertension status with AOBPM relative to daytime ABPM among the study cohort. Central pie chart represents the proportion of uncontrolled hypertension and normotension/controlled hypertension based on mean daytime ABPM ≥135/85 mmHg or <135/85 mmHg, respectively. White coat hypertension (if on no antihypertensive medication) or white coat effect (if on antihypertensive medication) was defined as AOBPM ≥140/90 mmHg but mean daytime ABPM <135/85 mmHg. Masked hypertension (if on no antihypertensive medication) or masked uncontrolled hypertension (if on antihypertensive medication) was defined as AOBPM <140/90 mmHg but mean daytime ABPM ≥135/85 mmHg.

Abbreviations: ABPM, ambulatory blood pressure monitoring; AOBPM, automated office-based blood pressure measurement; BP, blood pressure; HTN, hypertension.
